# One's sex, sleep, and posttraumatic stress disorder

**DOI:** 10.1186/2042-6410-3-29

**Published:** 2012-12-31

**Authors:** Ihori Kobayashi, Nancy Cowdin, Thomas A Mellman

**Affiliations:** 1 Department of Psychiatry, Howard University, 530 College St. NW, Washington, DC, 20060, USA; 2 Department of Neuroscience, Georgetown University, Washington, DC, USA

**Keywords:** Posttraumatic stress disorder, PTSD, Sleep, Sex differences

## Abstract

Women are approximately twice as likely as men to develop posttraumatic stress disorder (PTSD) after trauma exposure. Mechanisms underlying this difference are not well understood. Although sleep is recognized to have a critical role in PTSD and physical and psychological health more generally, research into the role of sleep in PTSD sex differences has been only recent. In this article, we review both animal and human studies relevant to sex differences in sleep and PTSD with an emphasis on the roles of sex hormones. Sleep impairment including insomnia, trauma-related nightmares, and rapid-eye-movement (REM) sleep fragmentation has been observed in individuals with chronic and developing PTSD, suggesting that sleep impairment is a characteristic of PTSD and a risk factor for its development. Preliminary findings suggested sex specific patterns of sleep alterations in developing and established PTSD. Sleep maintenance impairment in the aftermath of trauma was observed in women who subsequently developed PTSD, and greater REM sleep fragmentation soon after trauma was associated with developing PTSD in both sexes. In chronic PTSD, reduced deep sleep has been found only in men, and impaired sleep initiation and maintenance with PTSD have been found in both sexes. A limited number of studies with small samples have shown that sex hormones and their fluctuations over the menstrual cycle influenced sleep as well as fear extinction, a process hypothesized to be critical to the pathogenesis of PTSD. To further elucidate the possible relationship between the sex specific patterns of PTSD-related sleep alterations and the sexually dimorphic risk for PTSD, future studies with larger samples should comprehensively examine effects of sex hormones and the menstrual cycle on sleep responses to trauma and the risk/resilience for PTSD utilizing various methodologies including fear conditioning and extinction paradigms and animal models.

## Review

Posttraumatic stress disorder (PTSD) is a psychiatric condition that develops in some individuals after experiencing events involving threat of injury, physical integrity, or death [1]. Approximately 8% of adults in the U.S. meet diagnostic criteria for PTSD at some point in their lives [2]. PTSD consists of three clusters of symptoms: re-experiencing (e.g., recurrent nightmares about trauma, flashbacks), avoidance (e.g., avoidance of trauma reminders), and hyperarousal (e.g., insomnia, hypervigilance) [1]. In addition to these potentially debilitating symptoms, individuals with PTSD are at greater risk for other mental and physical health problems including suicidal behaviors, cardiovascular diseases, and gastrointestinal diseases [1,3-5].

Women have a higher lifetime prevalence of PTSD (10% in women vs. 5% in men) as well as a greater risk of developing PTSD following trauma exposure (13-20% vs. 6-8%), even though men have a higher risk for exposure to traumatic events (51% vs. 61%) [2,6]. A possible explanation for this sex difference is women’s higher risk for exposure to traumatic events associated with a greater risk of developing PTSD such as rape and molestation [2]. However, a meta-analysis conducted by Tolin and Foa [7] revealed women’s higher susceptibility to PTSD even after controlling for type of trauma, especially following exposure to trauma more frequently experienced by men (e.g., accidents, nonsexual assaults). They, therefore, concluded that trauma type is not the only factor contributing to the women’s greater risk of developing PTSD [7]. However, this analysis by category of trauma may not be sensitive to differences in the nature of assaults. Women are more likely to be exposed to more persistent assaults such as in the case of domestic violence than men [8], which may have influenced the women’s greater susceptibility. Although the meta-analysis showed slightly higher risks for PTSD in women following exposure to combats, it is important to note that recent studies examining veterans of recent wars in Iraq and Afghanistan have revealed small sex differences in combat exposure and no differences in the risk of developing PTSD after controlling for levels of combat exposure [9,10].

Various psychosocial and biological factors have been hypothesized to contribute to the sex differences in the risk for PTSD (see Olff, Langeland, Draijer, & Gersons, 2007 [11] for a review). Sleep has been recognized to play a critical role in PTSD (see the Sleep in PTSD section) as well as other mental and physical conditions (see the next section). Despite this, the role of sleep in the sexually dimorphic risk for PTSD has only recently begun to be investigated. In this article, we first review the role of sleep in physical and psychological health and in PTSD, and then we review sex differences and the roles of sex steroids in PTSD and sleep. Finally we present limited available findings and perspectives in the area of sex differences in PTSD-related sleep disturbance.

### Sleep and physical and psychological health

Relationships between sleep and physical health have been documented in a number of prospective and cross-sectional studies. Long and short sleep durations in contrast to 7–8 hours of habitual sleep are associated with higher mortality rates [12] and higher risk for coronary heart disease related events [13] and weight gain [14]. Sleep has also been linked to the chronic activation of immune function [15,16]. Experimental sleep restriction increased secretion of interleukin 6 and tumor necrosis factor-α [17,18]. Elevated basal levels of these proinflammatory cytokines were also observed in obstructive sleep apnea patients [19].

Subjective sleep disturbances predicted the onset of depressive symptoms in a general population, the elderly with a history of depression, and patients with bipolar disorder [20-22]. In an elderly group that received treatment for depression, greater subjective sleep impairment, less stage 2 sleep (N2), greater rapid-eye-movement (REM) sleep and REM activity before treatment were associated with slower recovery [23]. In contrast, a prospective study examining individuals going through divorce found that lower eye-movement density during the first REM episode at the beginning of their divorce process predicted more depressive symptoms one year later [24].

### Sleep in PTSD

Difficulty falling and staying asleep and recurrent trauma-related nightmares are two of the 17 diagnostic criteria for PTSD [1]. Individuals with PTSD are more likely to report these sleep disturbances than trauma-exposed individuals without PTSD [25,26]. For example, 91% of Vietnam veterans and 73% of rape or attempted rape victims with PTSD reported sleep initiation or maintenance problems as opposed to 62.5% and 18% of similarly exposed comparison subjects without PTSD, respectively [25,27]. Similarly, 38 to 73% of individuals with PTSD across studies reported experiencing recurrent nightmares about trauma, whereas only 6 to 12% of trauma-exposed individuals without PTSD reported having nightmares [25,27,28]. These sleep disturbances were also observed in individuals at greater risk for PTSD. Prospective studies showed that insomnia immediately prior to trauma exposure and soon after trauma predicted the subsequent development of PTSD [29,30]. In addition, the presence of trauma-related nightmares within a month of serious injury predicted PTSD symptoms severity at 6-weeks and 1-year post-injury [31,32].

In contrast to the consistently reported subjective sleep disturbances in PTSD, results of studies using polysomnography (PSG) are mixed, with some studies not finding differences between individuals with and without PTSD and the other studies documenting PTSD-related sleep impairments [33]. Overestimation of sleep impairments by people with PTSD has been suggested as a possible explanation for the discrepancies between subjective and objective sleep in PTSD [34]; however, the overestimation hypothesis has not been supported by several studies that evaluated relationships between subjective and objective sleep in PTSD [35-37]. A meta-analytic review of PSG studies revealed small effect sizes indicating that people with PTSD have more stage 1 sleep (N1) and less slow wave sleep (SWS), collectively indicating shallower sleep, than those without PTSD [33]. More consistent PSG findings exist regarding REM sleep activity and fragmentation. The meta-analysis found a medium-size effect of increased REM eye-movement density in PTSD. Individuals with chronic PTSD have exhibited signs of REM sleep interruption including a greater number of entries to N1 or wake from REM sleep or an increased percentage of N1 or wake during REM periods [38,39]. REM sleep fragmentation in the aftermath of trauma may also predict or have a role in the development of PTSD. A prospective study found a greater number of REM periods and shorter duration of continuous REM segments within a month of serious injury in patients who subsequently developed PTSD compared to injured patients who did not develop PTSD despite similar overall sleep maintenance and duration [40].

In the past decade, quantitative electroencephalography analysis (qEEG) has begun to be utilized in the field of sleep research, and a limited number of qEEG studies have been conducted in PTSD. In a lab-based PSG study, Woodward and colleagues [41] found reduced low-frequency [i.e., delta (0.2 – 3.8 Hz) and theta (4 – 7.8 Hz)] spectral power during SWS in male combat veterans with PTSD as compared to male veterans without PTSD even though no group difference was found in the percentage of traditionally scored SWS. However, Germain and colleague’s home PSG study with small samples [n = 10 (3 men) for PTSD and 5 (2 men) for control groups] [42] found increased delta (0.2 - 4 Hz) power during the entire sleep period in violent crime victims with PTSD compared to healthy controls. Germain and colleagues suggested that this result indicates increased sleep pressure induced by chronic sleep disruption in PTSD. Beta activity is considered a marker of cortical arousal [43,44], and alterations of beta spectral power in PTSD has been reported. Increased beta power (20–32 Hz) during the entire sleep period in PTSD was observed in the home PSG study, but less beta power during REM sleep in veterans (N = 29, 1 woman) with chronic PTSD (n = 16) compared to veterans without PTSD (n = 13) was reported in a lab-based PSG study [45]. In recently trauma-exposed individuals, reduced relative beta power (14–32 Hz) during REM sleep was associated with more severe PTSD symptoms and nightmares [46].

A role for REM sleep alteration is suggested by animal models of PTSD sleep disturbance that utilize fear conditioning. Male mice and rats exhibited reduced REM sleep and total sleep time (TST) during light phases (when mice and rats normally sleep) immediately following the footshock training [47-50]. In addition, re-exposure to cues and contexts paired with footshocks induced alteration in REM sleep including reduced REM sleep and REM segments duration in rats and mice [47,51,52] for as long as 34 days after the training [53]. Genetic factors seem to influence the susceptibility to REM sleep alterations following shock training and re-exposure to conditioned cues. The greater REM sleep suppression following footshock training was observed in stress-reactive BALB/cJ mice compared to the less reactive C57BL/6 J mice [47,54]. Re-exposure to conditioned cues fragmented REM sleep in stress-sensitive Wistar-Kyoto rats, but consolidated REM sleep in less sensitive Wister rats [55].

In summary, subjective reports of insomnia and trauma-related nightmares are common among people with PTSD. These sleep disturbances were also reported immediately before and after trauma exposure by people who developed PTSD, suggesting sleep disturbances increase the vulnerability for PTSD. However, it is also possible that these associations are a consequence of common risk factors. Although PSG studies inconsistently documented disturbances in sleep initiation and maintenance and sleep depth in PTSD, disrupted REM sleep has more consistently been reported. REM sleep fragmentation was found in individuals with chronic PTSD as well as recently traumatized people who subsequently developed PTSD. Analogous alterations of REM sleep were observed in rats and mice following exposure to footshocks and re-exposure to cues previously paired with footshocks. Strain comparisons revealed genetic influence on susceptibility to REM sleep disturbances following exposure to footshocks and stress cues. These findings of animal and human studies suggest that REM sleep disruption is not only a characteristic of established PTSD, but also a contributor to the development of PTSD.

### Sex differences in sleep

Given the role of sleep in PTSD, it is possible that sex differences in sleep contribute to the increased prevalence in women. In the general population, women across the adult age span are more likely than men to report having sleep initiation and maintenance problems, as well as nightmares [56-59]. However, PSG studies of the general population and healthy volunteers have shown that women have better sleep continuity indexed by less wake after sleep onset (WASO), higher sleep efficiency (SE), and deeper sleep as indicated by a lower percentage of N1 and higher percentage of SWS [60-62]. Women experience fewer respiratory disturbances during sleep; however, the sex differences in sleep impairment persisted after controlling for sleep-related respiratory disturbances [60,61,63].

Sex hormones and their fluctuations throughout the menstrual cycle likely contribute to the paradoxical relationship between sex and subjective/objective sleep. Female sex hormones have sleep-promoting effects when they are exogenously administered. Administration of progesterone to men increased non-REM sleep, and estrogen replacement improved sleep initiation and maintenance in hypogonadal and perimenopausal women [64]. In contrast to the sleep promoting effects of female sex hormones, exogenous administration of testosterone to men induced obstructive sleep apnea symptoms and reduced TST. Testosterone replacement increased REM sleep in hypogonadal men [65,66].

Women report greater subjective sleep disturbances and lowered sleep quality during the late-luteal phase when both estrogen and progesterone are declining and menstruation when both the hormones are low compared to the mid-follicular phase when estrogen is rising and progesterone is low [67,68]. Objective sleep changes across the menstrual cycle have been examined in PSG studies with small samples of healthy, naturally cycling women (*Ns* = 5–18). Preliminary results of those studies include shallower sleep, longer sleep onset latency (SOL), and altered REM sleep mostly during the mid- or late-luteal phase when progesterone is high and estrogen is moderate (mid-luteal) or both hormones are decreasing (late-luteal) compared to the mid-follicular phase [69-71]. The findings related to REM sleep include lower percentage of REM sleep in the late and mid-luteal phase, shorter REM period duration in the mid-luteal phase, and longer REM latency in the late-luteal phase compared to the mid-follicular phase [69,71]. In addition, qEEG revealed increased spectral power of sleep spindle frequency in the late-luteal phase compared to the late-follicular phase [72] and increased spectral power of the upper sleep spindle frequency range (14.25 – 15.0 Hz) during non-REM sleep in the luteal phase compared to the follicular phase [70]. However, it is important to note that findings of PSG studies are not entirely consistent. Three PSG studies with small samples (*Ns* < 10) did not detect differences in indices of sleep initiation, maintenance, or depth between the follicular and luteal phases [69,73,74].

Effects of exogenous and endogenous sex hormones on sleep were also found in animals, mainly during dark periods when they are normally active. Intact female (estrous cycle not controlled) C57BL/6 J mice had more wake time and less non-REM sleep during dark periods, but not during light periods, compared to intact males [75]. The differences in either wake or non-REM sleep were not observed between gonadectomized male and female mice. In Sprangue-Dawley rats, intact males had more REM sleep compared to intact females (estrous cycle not controlled) in both light and dark periods, and ovariectomy increased REM sleep during dark periods, but not during light periods [76]. In contrast with human studies, sleep suppressing effects of estradiol and sleep promoting effects of testosterone were observed in mice, although these effects were observed mainly during dark periods. Estradiol administration to ovariectomized female C57BL/6 J mice increased wake time and reduced both non-REM and REM sleep during dark periods, but testosterone administration to castrated male mice reduced wake time and increased non-REM sleep during dark periods [77]. These effects were not observed during light periods. In both Sprangue-Dawley and C57BL/6 J mice, increased REM sleep during dark periods was observed during the diestrus phase when both estradiol and progesterone levels are low compared to other estrous phases [76,78], although results have varied depending on strains of animals. Estrous cycle-related change in sleep was not found in BALB/cJ mice, but reduced light period REM and non-REM sleep were found during the diestrus phase compared to other phases in C3H/HeJ mice [78].

In summary, women report greater subjective sleep disturbances than men; however, in PSG studies, women typically sleep better than men. Effects of sex hormones and the menstrual/estrous cycle on sleep have been found in both human and animals, although the directions and timing (i.e., active or sleep periods) of effects differ by species. In humans, exogenously administered estrogen and progesterone promoted sleep; however, in the luteal phase when progesterone levels are high, women had shallower and more disrupted sleep and decreased and more fragmented REM sleep compared to the follicular phase when progesterone is low. In rats and some strains of mice, sleep suppressing effects of exogenous and endogenous female sex hormones were observed during dark periods when the animals are normally active. These findings underscore the importance of taking into account effects of sex hormones and the menstrual cycle in investigation of sex differences in sleep. The differences in results of animal and human studies warrant caution in using animal models to examine effects of exogenous sex hormones on human nighttime sleep.

### Roles of Sex hormones in the development of PTSD

There have been only a few human and animal studies that explored the roles of sex hormones in PTSD. Those studies utilized fear-conditioning and fear-extinction paradigms, as impairment in a fear-extinction process has been hypothesized to be critical to the pathogenesis of PTSD [75]. Milad and colleagues [79] found that men and women in the early-follicular phase, when both estrogen and progesterone levels are low, had greater retention of extinction memory measured by skin conductance compared to women in the late-follicular phase when estrogen is high. In animal studies, male and ovariectomized female rats showed slower extinction than intact female rats [80], and female rats in the proestrus phase, when both estrogen and progesterone are high, exhibited less freezing behaviors at the end of fear conditioning training and during extinction [81]. These findings suggest that sex hormones play a role in the fear-extinction process. Similar to the effects of exogenous sex hormones on sleep, the direction of effects may be different between humans and animals; however, the differences in measures used in human and animal studies (i.e., skin conductance vs. freezing; extinction recall vs. fear conditioning training and extinction) make the comparison of results difficult. Further investigation using the same measures is needed to elucidate directions of the effects.

### Sex differences in PTSD-related sleep disturbances

Only a limited number of studies have directly compared men’s and women’s subjective or objective sleep in PTSD. A study examining subjective sleep in chronic PTSD did not find sex differences (190 women and 177 men) in sleep reported in a sleep questionnaire [82]. As mentioned earlier, fragmented REM sleep was observed in individuals with chronic PTSD and developing PTSD [38-40]. A preliminary finding of a PSG study with a small number of participants suggested greater REM sleep fragmentation indexed by higher number of REM segments in men with PTSD (*n* = 3) compared to women with PTSD (*n* = 14) [83]. This study did not examine sex differences in other sleep parameters. A secondary analysis of the aforementioned prospective study examining PSG sleep in the aftermath of serious injuries found that REM sleep fragmentation indexed by the shorter duration of REM segments soon after trauma was associated with the development of PTSD in both sexes [40,84]. However, the REM fragmentation might be more pronounced in women than men as the effect size was greater in women [Cohen’s *d* = −0.6 for men (*n* = 10 for PTSD, 12 for non-PTSD groups), *d* = −1.1 for women (*n* = 6 for PTSD, and 7 for non-PTSD groups)]. The secondary analysis found sex differences in indices of overall sleep maintenance. Women who developed PTSD had longer WASO and less TST compared to men who developed PTSD and women who did not develop PTSD, respectively. Contrary to expectations, better sleep continuity indexed by shorter WASO was found in men who developed PTSD compared to men who did not develop the disorder. As both the PSG studies examining sex differences in established and developing PTSD had a small number of participants, results should be considered preliminary.

The aforementioned meta-analysis of PSG studies performed subgroup analyses with studies consisting of only male participants (13 studies) [33]. Results revealed less TST and SWS, more N1, and longer SOL in PTSD compared to controls. Only two studies have investigated PTSD-related objective sleep impairment with only female participants, and those studies revealed less TST and N2 measured by PSG and longer SOL and more movements measured by actigraphy in women with PTSD compared to women without PTSD [36,85]. However, these apparent PTSD-related sleep alterations found in the studies with only men and only women could be influenced by other sex-related factors such as the use of substances. Roles of sex hormones or the menstrual cycle in PTSD-related sleep impairment have not been investigated in human or animal studies.

In summary, only a few studies with a small number of participants have directly investigated sex differences in PTSD-related sleep alteration. Although fragmented REM sleep in the aftermath of trauma was associated with the development of PTSD in both sexes, the association might be stronger in women. Sleep maintenance impairment soon after trauma might be associated with the development of PTSD in women. Results of studies examining objective sleep of either men or women have revealed differences and similarities in PTSD-related sleep disturbances between sexes. Reduced deep sleep has been found in studies with only men, and impaired sleep initiation and maintenance with PTSD have been found in both studies with only men and only women. The role of sex hormones and the menstrual cycle on the PTSD-related sleep disturbances have not been studied.

## Conclusions

Research of chronic and developing PTSD has revealed that sleep impairment is a characteristic of PTSD and a risk factor for the development of PTSD, although the associations may also be related to common risk factors. Sex differences in the risk of developing PTSD following trauma exposure have been well documented; however, investigation into roles of sleep in the sex difference has only recently begun. Although recent preliminary evidence points to sex specific patterns of sleep impairment related to chronic and developing PTSD, mechanisms connecting the sex specific patterns and the development and maintenance of PTSD have not been investigated. The effects of sex hormones and the menstrual cycle on sleep and PTSD add complexity to the efforts to elucidate the mechanisms. Investigation of the roles of sex hormones in the development of PTSD and post-trauma sleep in trauma-exposed individuals entails methodological challenges (e.g., recruitment of recently traumatized individuals and measuring sex hormones and monitoring the menstrual cycle in the aftermath of trauma) and has not been conducted. The use of fear-conditioning and extinction paradigms may circumvent some of the methodological challenges and will yield information leading to further understanding of the roles of sex hormones in the development of PTSD. Animal PSG studies showed that exposure to footshock stress reduced REM sleep and the duration of REM segments [47,51,52]. Involvement of sex hormones in the fear extinction process was also suggested in animal and human fear-conditioning studies using skin conductance and freezing behaviors as outcome measures, respectively [79-81]. Future studies should examine effects of sex hormones and the menstrual/estrous cycle on sleep responses to fear-conditioning and extinction processes. Incorporation of neurohistological, neuroimaging, and qEEG methods in those studies would further elucidate mechanisms underlying sex differences in sleep and PTSD.

## Abbreviations

PTSD: Posttraumatic stress disorder; PSG: Polysomnography; REM: Rapid eye movement; EEG: Electroencephalogram; qEEG: Quantitative electroencephalography analysis; WASO: Wake after sleep onset; SOL: Sleep onset latency; SE: Sleep efficiency; TST: Total sleep time; TIB: Time in bed; SWS: Slow wave sleep; N1: Stage 1 sleep; N2: Stage 2 sleep.

## Competing interests

The authors declare that they have no competing interests.

## Authors’ contributions

IK reviewed the literature and drafted the manuscript. NC helped to review the literature and draft the manuscript. TAM helped to conceptualize and draft the manuscript. All authors read and approved the final manuscript.
